# Chronotype and psychological distress among Chinese rural population: A moderated mediation model of sleep quality and age

**DOI:** 10.1371/journal.pone.0241301

**Published:** 2020-10-30

**Authors:** Tianya Hou, Fan Zhang, Xiaofei Mao, Guanghui Deng

**Affiliations:** Department of Psychology, Naval Medical University (Second Military Medical University), Shanghai, China; Medical University of Vienna, AUSTRIA

## Abstract

**Purposes:**

Evidence suggests evening-type individuals have a higher risk of reporting psychological distress than morning-type individuals. However, less is known regarding the underlying processes that might mediate or moderate this association among Chinese rural population. This study aimed to evaluate the prevalence of psychological distress, investigate whether sleep quality would mediate the association between chronotype and psychological distress and explore whether age would moderate the direct or indirect effect of the mediation model.

**Methods:**

The cross-sectional study utilized a sample of 884 rural residents from rural regions in Anqing City, Anhui Province, China. Morningness-Eveningness Questionnaire (MEQ), Pittsburgh Sleep Quality Index (PSQI) and 21-item Depression Anxiety Stress Scale (DASS-21) were used to measure chronotype, sleep quality and psychological distress, respectively. MacKinnon’s four-step procedure was employed to examine the mediation effect, while Hayes PROCESS macro (model 59) was used to perform the moderated mediation analysis.

**Results:**

The prevalence of psychological distress among Chinese rural population was 33.4%. The association between chronotype and psychological distress was partially mediated by sleep quality (indirect effect = - 0.05, 95% CI = [-0.08, -0.03]). In addition, age moderated the first stage (sleep quality–psychological distress) of the indirect effect, with the indirect effect being attenuated for older rural residents. As suggested by Johnson-Neyman technique, the association between sleep quality and psychological distress was only significant when the age of the participant was lower than 48.59.

**Conclusions:**

The incidence of psychological distress among Chinese rural residents cannot be neglected. Interventions for the enhancement of sleep quality to prevent and reduce psychological distress should be prioritized to rural residents who are prone to eveningness, especially those who are younger.

## Introduction

With the sustained rapid social and economic development, there has been an increasing focus on psychological distress [1]. Psychological distress is a broad term that refers to a non-specific syndrome with anxiety and depression posited as the core symptoms [2, 3], and as the level of psychological distress enhances, the likelihood of the person with symptoms capable of meeting the diagnostic criteria of mental disorders increases [4]. The comorbid association between psychological distress and physical conditions could lead to poorer quality of life, reduced workplace productivity, increased utilization of healthcare resources, higher healthcare costs, and prolonged disability [4–7]. The prevention of psychological distress beforehand is a priority agenda for health.

The disruption of circadian rhythms is regarded as a risk factor in various mood-related problems encompassing depression and anxiety [8]. Previous literature suggested circadian clock-related genes might predispose individuals to anxiety disorders [9]. Likewise, the changes in the secretion of diurnal cortisol have been presented to be implicated in anxiety [10, 11] and the decrease in melatonin has been linked to affective disorders [12]. In addition, participants from anxiety and depression groups were more likely to report delayed sleep onset and offset time than those from the control group [13]. Chronotype as an indicator of circadian rhythms represents a continuous spectrum ranging from eveningness to morningness [14, 15]. Evening-type individuals are more active and perform better in the evening than in the morning and circadian processes (such as cortisol and body temperature) peaks in the evening, while it is vice versa for morning-type individuals [16]. More importantly, evening-type individuals are more likely to suffer from the desynchrony between circadian rhythms and environmental demands [14]. Considerable studies supported the evidence for the relation between eveningness and mood fluctuation based on healthy population [17, 18]. Thus, it is highly possible chronotype is linked to psychological distress in non-clinical samples.

Compared with urban residents, rural dwellers are more likely to be socio-economically disadvantaged and less educated, live in poorer social and physical environment, and have less access to health care services [19–21], which makes them more vulnerable to psychological distress. In China, the prevalence of depression in rural areas was 1.5 times higher than that in urban areas [22]. Additionally, there was a big difference in the occupational routines of the rural and urban populations [23]. For rural residents, there was no great disparity in the starting and finishing time of work. Furthermore, rural residents reported less recreational and communicative leisure activities than their urban counterparts [24]. Thus, the work obligations and the participation in leisure activities might also exert a regulatory effect on circadian rhythm among rural residents [25]. Although there are great discrepancies in lifestyles between urban and rural residents, the majority of previous literature on the associations between chronotype and psychological distress was only based on urban areas. To date, no study has explored the effect of chronotype on psychological distress in rural China. Given that rural residents accounted for approximately 43% of the total population in China [26], ongoing efforts are in need to further explore the association between chronotype and psychological distress among rural dwellers in China. In addition, there is an enormous explanatory gap in potential mechanisms underlying the association, which is vital to advance our understanding of psychological distress among Chinese rural dwellers.

### The mediating role of sleep quality

Accumulating evidence is emerging regarding the relation between chronotype and quality of sleep [27, 28] due to social jetlag which is defined as the misalignment of social time and chronotype [29]. Social jetlag presents to be higher in evening-type individuals. Previous study claimed evening chronotype was the most influential risk factor for poor quality of sleep [30]. Additionally, sleep quality is essential for optimal physical and psychological health [31]. Prior research showed the negative association between sleep quality and psychological distress [32]. According to the literature review, sleep quality might mediate the association between chronotype and psychological distress among Chinese rural dwellers.

### The moderating role of age

Existing literature has indicated the age differences in the association between chronotype and depressive symptoms among a rural population, suggesting a more prominent relation in participants aged 31–40 years [33]. Nevertheless, a more previous study reported the totally different findings that the association between chronotype and depressive symptoms was stronger in younger (aged≤ 20 years) or older (aged ≥50 years) participants [34]. Despite the inconsistent findings from literature review, they reached the consensus on the moderating role of age in the mechanisms through which chronotype influences psychological distress. A meta-analysis conducted by Au and Reece [35] summarized 36 studies and revealed the research concerning the moderating role of age in the relation between chronotype and mood symptoms was too few to ascertain the relationship.

### The present study

**Fig 1:**
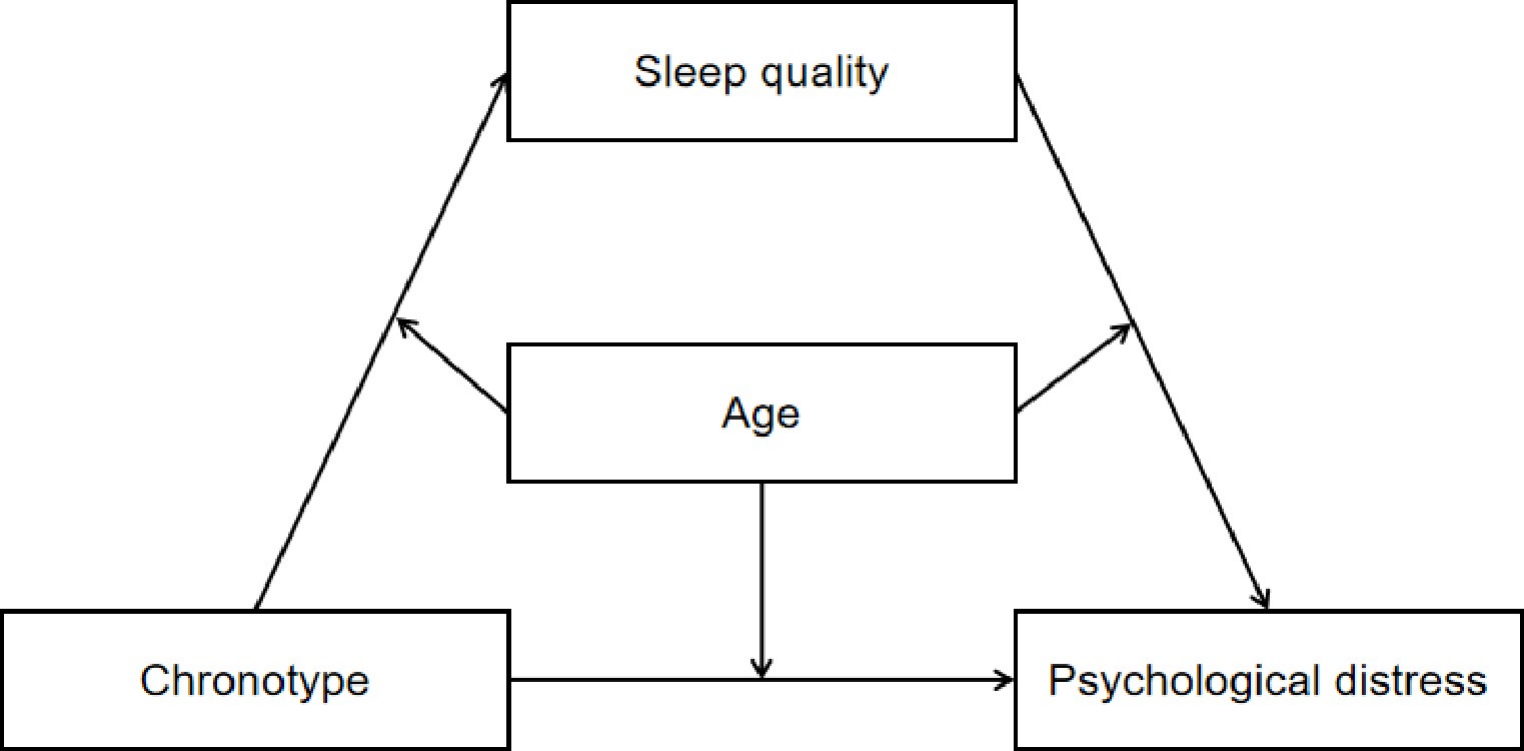
Conceptual model.

The primary objective of the present study was to estimate the prevalence of psychological distress among rural dwellers in rural China, while the second objective was to shed light on “how” and “for whom” chronotype would be associated with psychological distress through a moderated mediation model (see **Fig 1**). We hypothesized that sleep quality might serve as a mediator to bridge the link between chronotype and psychological distress. In addition, age would moderate the direct and/or indirect effect (including chronotype—sleep quality path and sleep quality–psychological distress path) of chronotype on psychological distress.

## Methods

### Participants and procedures

The cross-sectional study was conducted in the rural regions of Anqing City, Anhui Province, China, from December, 2019 to January, 2020. Participants were recruited through cluster sampling procedures. The inclusion criteria were a) aged between 18 and 54, and b) having a rural Hukou. The exclusion criteria were: a) participants with missing values on any of the dependent, independent and control variables, b) those whose answer time was less than 8 minutes and c) those who reported longer sleep duration than time in bed, which caused the sleep efficiency (sleep duration/time in bed) was more than 1. Finally, 884 participants were included in the analysis.

Before completing the survey, all participants were given informed written consent, which claimed the participation was voluntary and anonymous and could be withdrawn at any time. The research ethics committee of Naval Medical University approved the study.

### Measures

#### Sociodemographic characteristics

Self-reported age, gender, marital status, educational level, the status of having siblings and family structure were collected. Marital status was grouped into unmarried (single, divorced and widowed) and married. Educational level was categorized as primary school or below, junior high school, and senior high school or above. Family structure was classified into two groups: intact and non-intact.

#### Chronotype

Chronotype was assessed by Morningness-Eveningness Questionnaire (MEQ) developed by Horne and Ostberg [36], which is the most widely used instrument for the identification of circadian preference [37]. The total score of 19-item scale ranges from 16 to 86 points, with a higher score denoting the tendency to morning activities. The Chinese version scale has been consolidated as a reliable and valid instrument [38, 39].

#### Sleep quality

The Chinese version of Pittsburgh Sleep Quality Index (PSQI) was used to measure sleep quality [40]. Although the scale contains 24 items, only 19 items are used to calculate sleep quality. The 19 questions scored on a 3-point Likert scale in the reference period of “past 1 month” were grouped into 7 dimensions: subjective sleep quality, sleep latency, sleep duration, sleep efficiency, sleep disturbances, use of sleep medications and daytime dysfunction. The total score is generated by the sum of the scores of the 7 components ranging from 0 to 21 with a lower score indicating higher sleep quality. The Chinese version of PSQI has shown impressive reliability and sufficient validity [41].

#### Psychological distress

Psychological distress was measured by 21-item Depression Anxiety Stress Scale (DASS-21) developed by Lovibond and Lovibond [42] in its Chinese version, which is a common instrument for screening emotional distress symptoms during the past week [43]. The scale contains three subscales: Depression, anxiety and stress. Each subscale consists of 7 items and participants rated each item on a 4-point Likert scale from 0 (“did not apply to me at all”) to 4 (“apply to me very much”). The range of the total scores for each subscale is 0–42. Zanon and colleagues [44] based on eight countries including China examined the dimensionality of DASS-21 and suggested that DASS-21 would be best utilized as a unidimensional distress scale rather than three separate components of depression, anxiety and stress. In addition, the combination of 21 items presented the optimal sensitivity (79.1%) and specificity (77.0%) at the cut-off point of ≥34 of the total scale to screen psychological distress compared with any of the subscales or the combination of any two subscales [45]. The Chinese version scale has presented excellent reliability and good validity [46].

### Statistical analysis

Descriptive statistics, independent *t*-test and one-way analysis of variance (ANOVA) were performed for the description of demographic characteristics and group comparisons of MEQ, PSQI and DASS-21 scores. Pearson correlation analyses were conducted to calculate the bivariate correlations between the variables of interest. Then, the current study examined the mediation effect through MacKinnon’s four-step procedure [47]. Four criteria need to be satisfied to verify the mediation effect. Firstly, the association between chronotype and psychological distress would be significant. Secondly, the relation between chronotype and sleep quality would be significant. Thirdly, the relationship between sleep quality and psychological distress would still be significant when controlling for chronotype. Lastly, the coefficient for the indirect pathway between chronotype and psychological distress via sleep quality would be significant, which would be tested by the bias-corrected percentile bootstrap method using Hayes’s [48] PROCESS macro (Model 4). The last condition would be met if the 95% bias-corrected confidence interval (CI) calculated with 5000 resamples excluded zero. Finally, Model 59 would be performed to test the moderated mediation model, that was, whether age would moderate the direct and the indirect effects of chronotype on psychological distress. Likewise, if the 95% bias-corrected CI of the interaction did not include zero, the moderating effect of age would be established. Bootstrap method would be conducted to reveal the conditional effect at different values of age (1 SD below the mean, at the mean, and 1SD above the mean). Furthermore, we plotted the conditional effects and confidence bonds according to Johnson-Neyman technique [49]. All statistical analyses were performed by SPSS 25.0 and a two-tailed *P*-value less than 0.05 indicated statistical significance. All the study variables were standardized and all models were controlled for gender, marital status, the status of having siblings, educational level and family structure.

## Results

### Demographic characteristics and psychological distress

In the present study, 884 participants from rural areas had a mean age of 36.7 (SD = 7.1), ranging from 18 to 54. **Table 1** shows the detailed information. Most participants were females (63.0%), married (92.9%), having siblings (95.7%), reported junior high school education (57.8%) and intact family structure (87.0%). The prevalence of psychological distress among rural dwellers was 33.4%. According to the comparison of the means, males presented higher DASS-21 scores than females (*t* = 17.02, *P* < 0.001). Significant differences in MEQ and DASS-21 scores were found across marital status (*t* = 6.18, *P* = 0.013 and *t* = 6.98, *P* = 0.008). Educational level has a significant effect on MEQ scores (*t* = 8.39, *P* < 0.001). LSD post hoc test presented that participants with an educational level of primary school or below had higher MEQ scores than the other two groups (junior high school: mean difference = 1.43, *P* = 0.011; senior high school or above: mean difference = 2.61, *P* < 0.001). Those who had an educational level of junior high school presented higher scores of MEQ than the senior high school or above group (mean difference = 1.19, *P* = 0.016).

**Table 1:** Demographic characteristics and group comparisons of MEQ, PSQI and DASS-21 scores (n = 884).

	No. (%)	MEQ Scores		PSQI Scores		DASS-21 Scores	
		M ± SD	*P*	M ± SD	*P*	M ± SD	*P*
Gender			0.156		0.771		**<0.001**
Male	327 (37.0)	62.1 ± 5.8		4.9 ± 3.0		32.9 ± 29.7	
Female	557 (63.0)	61.5 ± 6.3		4.8 ± 3.1		25.4 ± 23.5	
Marital status			**0.013**		0.237		**0.008**
Unmarried	63 (7.1)	59.9 ± 7.4		5.3 ± 3,2		36.5 ± 28.7	
Married	821 (92.9)	61.8 ±6.0		4.8 ± 3.1		27.5 ± 25.9	
The status of having siblings			0.651		0.679		0.376
No	38 (4.3)	61.3 ± 5.1		4.7 ± 3.3		31.8 ± 26.0	
Yes	846 (95.7)	61.7 ± 6.2		4.9 ± 3.1		28.0 ± 26.2	
Educational level			**<0.001**		0.316		0.700
Primary school or below	155 (17.5)	63.2 ± 5.5		5.2 ± 3.0		27.7 ± 23.3	
Junior high school	511 (57.8)	61.7 ± 6.1		4.8 ± 3.0		27.7 ± 25.7	
Senior high school or above	218 (24.7)	60.6 ± 6.3		4.8 ± 3.3		29.5 ± 29.2	
Family structure			0.999		0.717		0.259
Intact	769 (87.0)	61.7 ± 6.2		4.8 ± 3.1		28.5 ± 26.8	
Non-intact	115 (13.0)	61.7 ± 5.9		5.0 ± 3.1		25.6 ± 21.7	

### Bivariate analyses

**Table 2** presents the means, SD and correlations between the study variables. The results showed that chronotype was negatively correlated with PSQI scores, DASS-21 and its three subscales (all *P* < 0.05), and positively associated with age (*P* < 0.001). PSQI scores were positively related to DASS-21 and its three subscales (all *P* < 0.001).

**Table 2:** Bivariate correlations among chronotype, PSQI scores, age, psychological distress and its subscales (n = 884).

	Mean	1	2	3	4	5	6
1. Chronotype	61.7 ± 6.1						
2. PSQI scores	4.9 ± 3.1	-0.239***					
3. Age	36.7 ± 7.1	0.219***	0.004				
4. Depression subscale of DASS-21	7.7 ± 9.4	-0.124***	0.173***	-0.023			
5. Anxiety subscale of DASS-21	9.3 ± 9.0	-0.080*	0.189***	-0.014	0.879***		
6. Stress subscale of DASS-21	11.2 ± 9.0	-0.137***	0.268***	-0.023	0.857***	0.879***	
7. DASS-21	28.2 ± 26.2	-0.119***	0.219***	-0.021	0.955***	0.961***	0.953***

*P<0.05.

**P<0.01.

***P<0.001.

### Testing for mediation effect

As multiple regression analysis showed, chronotype was negatively associated with psychological distress (*β* = -0.13, *P*<0.001) (see Model 1 in **Table 3**). Chronotype was also negatively correlated with PSQI scores (*β* = -0.27, *P*<0.001) (see Model 2 in **Table 3**), which indicated chronotype positively predicted sleep quality. In addition, PSQI score was positively associated with psychological distress while controlling for chronotype (*β* = 0.20, *P*<0.001) (see Model 3 in **Table 3**), which showed the negative association between sleep quality and psychological distress was significant when controlling for chronotype. Finally, the results of bias-corrected percentile bootstrap method presented that the indirect path between chronotype and psychological distress via PSQI scores (sleep quality) was significant (ab = -0.05, *SE* = 0.01, 95% CI = [-0.08, -0.03]). The mediation effect explained 39.9% of the total effect. Taken together, all four conditions of MacKinnon’s four-step procedure (2008) were satisfied, which supported the mediation effect.

**Table 3:** Mediation analysis (n = 884).

Variables	Model 1(Psychological distress)		Model 2 (PSQI Scores)		Model 3(Psychological distress)	
	*β*	*t*	*β*	*t*	*β*	*t*
Chronotype	-0.13***	-3.70	-0.27***	-7.57	-0.08*	-2.20
PSQI Scores					0.20***	5.88
R^2^	0.04***		0.07***		0.08***	
F	5.42		8.82		9.25	

*Note*: controlling for gender, marital status, the status of having siblings, educational level and family structure.

**P<0.01.

***P<0.001.

### Testing for moderated mediation effect

The results of the moderated mediation model are presented in **Table 4**. We expected that age would moderate the association between chronotype and psychological distress either both or separately in the direct effect (chronotype–psychological distress) and indirect effect (path a: chronotype–sleep quality and path b: sleep quality and psychological distress). Nonetheless, age did not moderate the direct effect of chronotype on psychological distress (chronotype * age: *β* = 0.040, 95% CI = [-0.169, 0.090]). The results revealed that only the first stage of the indirect effect of chronotype on psychological distress through sleep quality (PSQI scores) was moderated by age (chronotype–PSQI scores: *β* = 0.066, 95% CI = [0.006, 0.260]; PSQI scores–psychological distress: *β* = -0.008, 95% CI = [-0.121, 0.106]), indicating that the mediation effect of sleep quality on the relation between chronotype and psychological distress was moderated by age among the Chinese rural dwellers.

**Table 4:** Conditional process analysis (n = 884).

	*β*	*SE*	LLCI	ULCI
Mediator variable model (Outcome: PSQI scores)				
Chronotype	-0.264***	0.036	-0.334	-0.194
Age	0.066	0.068	-0.067	0.199
Chronotype * Age	0.066*	0.065	0.006	0.260
Dependent variable model (Outcome: Psychological distress)				
Chronotype	-0.075*	0.036	-0.146	-0.003
PSQI score	0.198***	0.034	0.132	0.264
Age	-0.053	0.067	-0.185	0.079
Chronotype * Age	-0.040	0.066	-0.169	0.090
PSQI scores * Age	-0.008	0.058	-0.121	0.106
	*β*	Boot *SE*	BootLLCI	BootULCI
Conditional indirect effect analysis				
1 SD below the mean	-0.068	0.019	-0.108	-0.034
Mean	-0.052	0.011	-0.076	-0.032
1 SD above the mean	-0.037	0.013	-0.065	-0.014

Outcome variable: Psychological distress

Note: controlling for gender, marital status, the status of having siblings, educational level and family structure.

* *p*<0.05

** *p*<0.01

*** *p*<0.001

**Fig 2:**
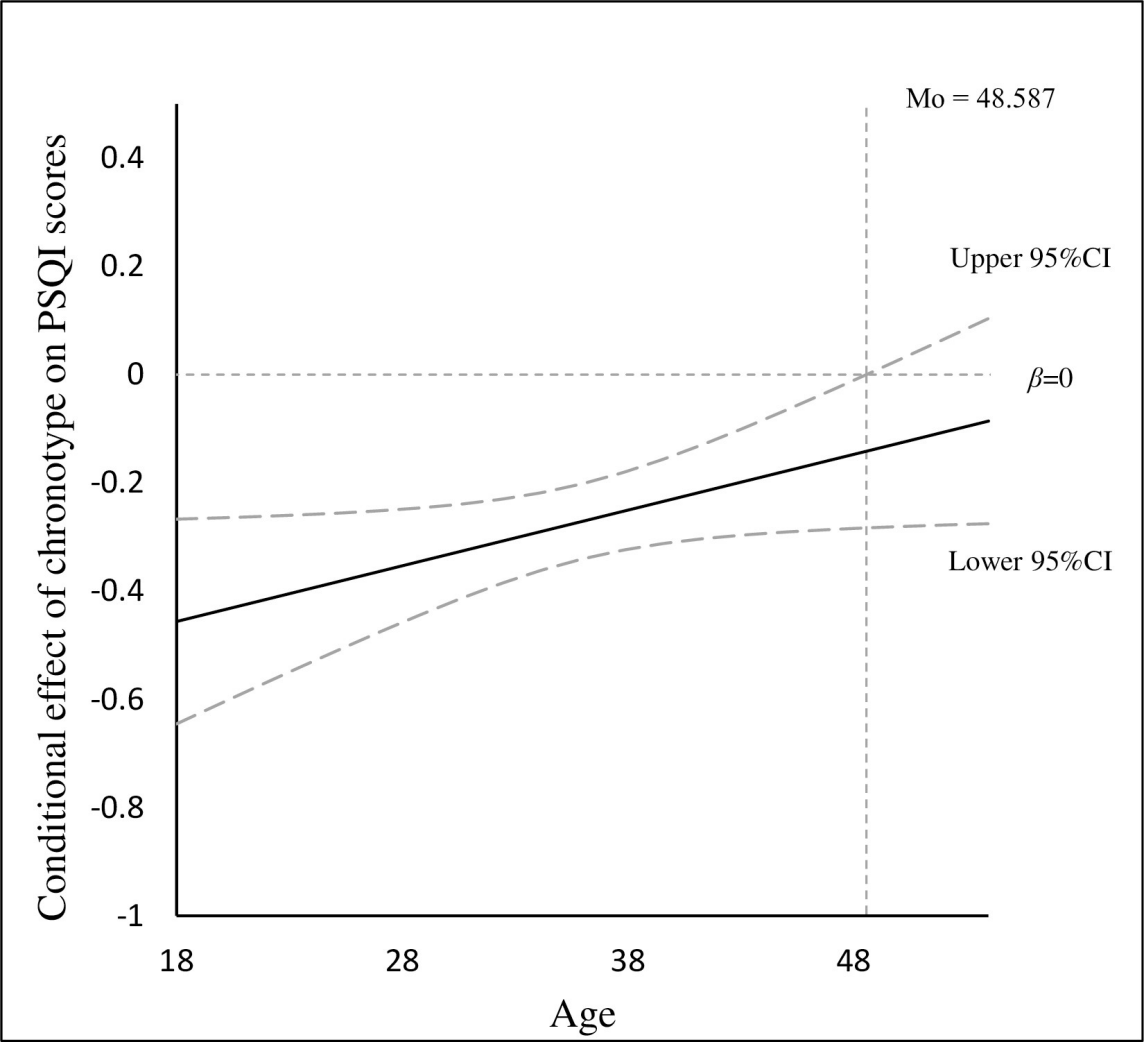
The conditional effect of chronotype on PSQI scores.

The conditional indirect effect of chronotype on psychological distress via sleep quality (PSQI scores) at different levels of age was performed by bootstrap method. The indirect effect was evaluated at 1 SD below the mean, the mean, and 1SD above the mean (see **Table 4**). The mediation effect of sleep quality on the association between chronotype and psychological distress became attenuated at 1SD above the mean of age (*β* = -0.037, 95% CI = [-0.065, -0.014]) than 1SD below the mean of age (*β* = -0.068, 95% CI = [-0.108, -0.034]). Moreover, the Johnson-Neyman technique (see **Fig 2**) showed that age could moderate the association between chronotype and sleep quality (PSQI scores) when the age of the participant was lower than 48.59 since the 95% confidence interval did not contain zero.

## Discussion

In the present research, the prevalence of psychological distress among rural residents from Anhui Province was 33.4%, which is slightly higher than that (33%) of the rural residents in Shangdong Province, China and that (31%) in rural communities in Australia [50, 51]. Earlier literature showed that the rates of psychological distress for famers and non-farmers in rural China were 31.13% and 30.01%, respectively [52]. The discrepancy might be attributed to different measurements and regional differences. In addition, the literature regarding the prevalence of psychological distress among urban population was 15.8–27.8% [53–55]. This is consistent with the previous study regarding the difference in psychological distress between urban and rural regions based on 9 countries, which suggested participants living in village presented higher odds of suffering from psychological distress than those in the cities [56]. Moreover, prior study reported the population-level prevalence of psychological distress in Australia and Canada were 11.1% and 12.0%, suggesting lower rates than our result. This indicated that compared with developed countries, Chinese rural population might be more likely to suffer from psychological distress on account of low income, educational level and poor mental health services [52]. In sum, the prevalence of psychological distress among rural Chinese residents could not be neglected and more attention should be paid to address this issue.

The current study showed males had significant higher scores of psychological distress than females, which contradicted with existing findings [57–60]. The participants in the current analysis were those with rural Hukou from Anhui Province where considerable rural residents migrated to seek work [61]. According to National Bureau of Statistics of China [62], the majority of rural migrant workers were males, accounting for 65.2% of the total migrant workers. Thus, males had a higher tendency to suffer from prolonged working hours, poor living circumstance, experiences of stigma and social inequity [63, 64], which indicated they were more likely to experience distress. Furthermore, this might also be attributed to the cultural value of work. In traditional Chinese society, males were anticipated to play the role of financial supporters of the whole family, and male migrant workers were more likely to expect or be expected to send money back home [61]. All these might explain why males presented higher levels of psychological distress than females in rural China. The present study also showed that unmarried rural residents presented higher levels of psychological distress compared to married residents. This is consistent with the previous findings [65]. Married people have more social support, which plays a critical role in the mitigation of negative emotions. Our results also suggested married rural dwellers and those with lower levels of education presented to be prone to morningness.

Consistent with previous studies [8, 66], the results found the significant association between chronotype and psychological distress among rural residents. This could be explained by Social Zeitgeber Theory [67]. Stressful life events would result in the alterations in the social zeitgebers including sleep-wake schedules. This would contribute to the change of molecular and cellular rhythms, which might further trigger the onset of negative emotions. As mentioned earlier, CLOCK gene variations might exert an influence on the structure of brain, which might be associated with both circadian regulation and emotion [68]. In addition, a recent study conducted by Horne and Norbury [69] revealed that eveningness was related to the increase of amygdala response to negative faces and was more likely to suffer from impaired emotion regulation. Thus, evening-type rural residents might be more likely to report psychological distress.

### The mediating role of sleep quality

The study also found sleep quality of rural residents partially meditated the association between chronotype and psychological distress, which was in line with our hypothesis and previous literature [70]. Although Smidt and colleagues [71] reported opposite findings, the research only had 52 participants and only abstract was published. Therefore, the quality of the research might be questioned. Evening-type individuals have a higher tendency to drink caffeinated beverage, eat snacks at midnight and suffer from internet addiction [72, 73]. A recent study reported the problematic smartphone use contributed to the enhanced risk of poor sleep quality, anxiety and depression [74]. Another study provided support regarding the positive correlations between PSQI scores and DASS-21 based on a non-clinical sample [75]. In sum, rural dwellers who were prone to eveningness tended to have poorer sleep quality, which further resulted in psychological distress. Thus, enhancing sleep quality might help reduce psychological distress among rural residents, especially those who are prone to eveningness. Prior literature has documented that sleep quality would benefit from exercise, mindfulness and music therapy [76–78], which would further contribute to the decrease in psychological distress.

### The moderating role of age

The results presented that age only moderated the association between chronotype and sleep quality among Chinese rural residents. Our findings suggested the association was attenuated in older rural dwellers than younger rural dwellers. As Johnson-Neyman technique presented, with the increase in age, the association between chronotype and sleep quality became weakened. When the age of the participants enhanced to 48.59 and over, the effect of chronotype on sleep quality became insignificant. Sleep problems are more normative as getting older [79] since there were many other influential factors that could disturb sleep, such as the loss and atrophy of neurons, reduced cerebral blood flow and neurotransmitter defects [80]. Thus, the effect of chronotype on sleep quality became weakened for middle-aged rural residents in comparison to younger rural residents.

Contrary to our expectations, age did not moderate the direct effect of chronotype on psychological distress and the association between sleep quality and psychological distress. This expanded the previous literature by uncovering age moderated the relationship between chronotype and psychological distress through moderating the first stage (chronotype—sleep quality) of the indirect path. Taken together, the moderated mediation model has profound implications. The interventions regarding the improvement of sleep quality should be prioritized for rural residents who are prone to eveningness and younger.

### Limitations and strengths

Some limitations must be addressed. Firstly, we employed a cross-sectional design, which cannot infer the causal relationship. In the future, longitudinal research could be conducted to validate the results of our study. Secondly, research data were collected through participants’ self-report, which might be subjective and lead to self-reported bias. Further study might benefit from collecting data from various informants (e.g., friends). Thirdly, the participants in our study were all from the rural area of Anhui province, which might geographically limit a wider generalization. Further research could collect data from various regions to ensure a broader generalization. Lastly, psychological distress as a complex concept could be influenced by considerable factors. Only part of psychological distress could be explained by chronotype and sleep quality. Also, there are other variables possibly explaining the associations between chronotype and psychological distress, such as emotional processing [81] and clock genes [82]. A more integrated model with more variables is suggested for further investigation in order to more comprehensively understand psychological distress among rural population.

Despite several limitations, the study adds knowledge to the previous literature, both theoretically and practically. Theoretically, the study sheds light on the potential mechanisms underlying the association between chronotype and psychological distress with the mediating and moderating effects of sleep quality and age, respectively. Practically, the study enlightens the interventions for the prevention and reduction of psychological distress among rural residents.

## Conclusion

To our knowledge, this is the first study to explore the relationship between chronotype and psychological distress among Chinese rural population through a moderated mediation model. The prevalence of psychological distress was 33.4% in the study. Sleep quality mediated the association between chronotype and psychological distress. Additionally, age moderated the strength of the mediating effect with the indirect association being attenuated in older Chinese rural residents. For Chinese rural residents who are prone to eveningness, especially those who are younger, it might be essential to design the interventions concerning the enhancement of sleep quality to prevent and decrease psychological distress.

## Supporting information

S1 Dataset (ZIP)
